# Identification of Novel Tumor-Associated Cell Surface Sialoglycoproteins in Human Glioblastoma Tumors Using Quantitative Proteomics

**DOI:** 10.1371/journal.pone.0110316

**Published:** 2014-10-31

**Authors:** François Autelitano, Denis Loyaux, Sébastien Roudières, Catherine Déon, Frédérique Guette, Philippe Fabre, Qinggong Ping, Su Wang, Romane Auvergne, Vasudeo Badarinarayana, Michael Smith, Jean-Claude Guillemot, Steven A. Goldman, Sridaran Natesan, Pascual Ferrara, Paul August

**Affiliations:** 1 Sanofi-Aventis Recherche & Développement, Centre de Toulouse, Toulouse, France; 2 ALS Therapy Development Institute, Cambridge, Massachusetts, United States of America; 3 Department of Neurology, University of Rochester Medical Center, School of Medicine and Dentistry, Rochester, New York, United States of America; 4 Sanofi US, Cambridge, Massachusetts, United States of America; 5 Sanofi Tucson Research Center, Oro Valley, Arizona, United States of America; University of Edinburgh, United Kingdom

## Abstract

Glioblastoma multiform (GBM) remains clinical indication with significant “unmet medical need”. Innovative new therapy to eliminate residual tumor cells and prevent tumor recurrences is critically needed for this deadly disease. A major challenge of GBM research has been the identification of novel molecular therapeutic targets and accurate diagnostic/prognostic biomarkers. Many of the current clinical therapeutic targets of immunotoxins and ligand-directed toxins for high-grade glioma (HGG) cells are surface sialylated glycoproteins. Therefore, methods that systematically and quantitatively analyze cell surface sialoglycoproteins in human clinical tumor samples would be useful for the identification of potential diagnostic markers and therapeutic targets for malignant gliomas. In this study, we used the bioorthogonal chemical reporter strategy (BOCR) in combination with label-free quantitative mass spectrometry (LFQ-MS) to characterize and accurately quantify the individual cell surface sialoproteome in human GBM tissues, in fetal, adult human astrocytes, and in human neural progenitor cells (NPCs). We identified and quantified a total of 843 proteins, including 801 glycoproteins. Among the 843 proteins, 606 (72%) are known cell surface or secreted glycoproteins, including 156 CD-antigens, all major classes of cell surface receptor proteins, transporters, and adhesion proteins. Our findings identified several known as well as new cell surface antigens whose expression is predominantly restricted to human GBM tumors as confirmed by microarray transcription profiling, quantitative RT-PCR and immunohistochemical staining. This report presents the comprehensive identification of new biomarkers and therapeutic targets for the treatment of malignant gliomas using quantitative sialoglycoproteomics with clinically relevant, patient derived primary glioma cells.

## Introduction

Glioblastoma multiform, a WHO grade IV astrocytoma that accounts for >53% of all gliomas, is the most frequent and devastating primary malignant brain tumor in adults. The mortality rate is very high, with a median survival in the range of 12–15 months post-diagnosis. In spite of advanced standard of care, namely maximal safe surgical resection, followed by radiation therapy plus concomitant and adjuvant chemotherapy with temozolomide, the survival rate is less than 5% [1]. Therefore, GBM remains an area of significant unmet medical need and innovative therapeutic strategies to eradicate residual tumor cells and prevent tumor recurrences are urgently needed. In contrast to conventional therapies, targeted toxins (immunotoxins or ligand-directed toxins) represent a new class of anticancer agents providing high specificity for tumor cells that selectively overexpress cell surface or extracellular matrix protein targets [2], [3]. In recent years, treatment of gliomas with targeted toxins has shown promising results both in animal models and in clinical trials [4], [5]. Examples of targeted toxins currently under study both in vitro and in preclinical models of HGGs are listed in Table 1. Several targeted toxins presented in Table 2, either as single agents or part of combination protocols, have shown impressive anticancer activity with acceptable profile of toxicity and safety in various stages of clinical development for the treatment of adult or pediatric patients with recurrent or progressive malignant glioma. These data strongly suggest that tumor specific antigens or receptors which are uniquely expressed at the surface of HGG cells but not by normal cells are excellent candidates for targeted therapies.

**Table 1:** Current in vitro and preclinical developments for investigational targeted toxins in glioblastoma.

Name	Component	Target	Indication	References
***Immunotoxins***				
9.2.27-PE	Anti-HMW-MAA/PE	HMW-MAA	Glioblastoma multiform	[*78*]
8H9scFV-PE38	Anti-B7H3 scFv/PE38	B7H3	High grade glioma	[*79*]
F6V-PE38	Anti-GPNMB scFv/PE38	GPNMB	Malignant glioma	[*80*]
7EAB11-SAP	Anti-PTPRZ1	PTPRZ1	Glioblastoma multiform	[*59*]
D2C7-(scdsFv)-PE38KDEL	Anti-EGFR/EGFRvIII scdsFv/PE38KDEL	EGFR & EGFRvIII	Glioblastoma multiform	[*81*]
NZ-1-(scdsFv)-PE38KDEL	Anti-podoplanin scdsFv/PE38KDEL	Podoplanin	Glioblastoma multiform	[*82*]
DmAb14m-(scFv)-PE38KDEL	Anti-3'-isoLM1/3',6'-isoLD1 scFv/PE38KDEL	3′-isoLM1 & 3′,6′-isoLD1	Glioblastoma multiform	[*83*]
***Ligand-directed toxins***				
DTAT	AT-uPA/DT	uPAR	High grade glioma	[*84*]
DT390IL13	IL-13/DT390	IL13RA2	Glioblastoma multiform	[*85*]
DAB389EGF	EGF/DT389	EGFR	Glioblastoma multiform	[*86*]
DTEGF13	IL-13/EGF/DT389	IL13RA2 & EGFR	High grade glioma	[*87*]
EGFATFKDEL 7mut	EGF/ATF/PE38KDEL	EGF & uPAR	High grade glioma	[*88*]
***Radioimmunotoxins***				
^125^I-labeled anti-MRP3 scFv	Anti-MRP3/^125^I	MRP3	Glioblastoma multiform	[*89*]

PE = whole *Pseudomonas* exotoxin; PE38 = truncated form of PE in which amino acids 1–252 and amino acids 365–380 are deleted; PE38KDEL = PE38 with a terminal lysyl-aspartyl-glutamyl-leucine sequence; DT = whole diphtheria toxin; DT389 & DT390 = first 389/390 amino acids of diphtheria toxin; AT-uPA & ATF = amino terminal fragments of urokinase-type plasminogen activator (uPA); uPAR = urokinase-type plasminogen activator receptor; HMW-MAA = high-molecular-weight melanoma-associated antigen; GPNMB = Glycoprotein NMB; EGF = epidermal growth factor; EGFR = epidermal growth factor receptor; EGFRvIII = mutant EGFR containing a deletion of 267 amino acids from the extracellular domain; IL-13 = Interleukin 13; IL13RA2 = interleukin 13 receptor, alpha 2; PTPRZ1 = receptor-type tyrosine-protein phosphatase zeta; 3′-isoLM1 and 3′,6′-isoLD1 = Glioma-associated foetal ganglioside antigens; NZ-1 = podoplanin.

**Table 2:** Current clinical trials for investigational targeted toxins in glioblastoma (HTTP://www.clinicaltrials.gov).

Name	Component	Target	Indication	Clinical phase	References
***Immunotoxins***					
^131^I-chTNT-1/B mAb	^131^I-anti-TNT/^131^I	DNA histone complex	Malignant glioma	I - II	[*90*]
^131^I-anti-TNC mAb 81C6	^131^I-anti-TNC/^131^I	Tenascin	Glioblastoma multiform	I/II - III	[*64*]
^211^At-anti-TNC mAb (81C6)	anti-TNC/^211^At	Tenascin	Recurrent malignant glioma	I - II	[*64*]
^125^I-anti-EGFR mAb 425	Anti-EGFR/^125^I	EGFR	Glioblastoma multiform	I/II	[*91*]
***Ligand-directed toxins***					
Tf-CMR107	Transferrin/DT	TfR	Glioblastoma multiform	I - III	[*92*]
TP-38	TGFα/PE38	EGFR	Glioblastoma multiform	II	[*93*], [*94*]
IL-4(38-37)-PE38KDEL	IL-4/PE38KDEL	IL4R	Recurrent malignant glioma	I - II	[*95*]
IL13-PE38QQR	IL-13/PE38QQR	IL13RA2	Recurrent malignant glioma	I - III	[*96*], [*97*]

PE38 = truncated form of *Pseudomonas* exotoxin in which domain Ia (amino acids 1–252) and amino acids 365–380 are deleted; PE38KDEL = PE38 with a terminal lysyl-aspartyl-glutamyl-leucine sequence; PE38QQR = PE38 in which lysine (K) residues are replaced by glutamine (Q) at positions 590 and 606 and by arginine (R) at position 613; DT = whole diphtheria toxin; IL-13 = Interleukin 13; IL13RA2 = interleukin 13 receptor, alpha 2; IL-4 = interleukin 4; IL4R = interleukin 4 receptor; TNT = tumor necrosis antigen; TGFα = transforming growth factor alpha; EGFR = epidermal growth factor receptor; TfR = transferrin receptor; TNC = tenascin.

In recent years, glioma proteomics aiming to identify biomarkers, grade specific signatures, and novel effective drug targets has been attempted at different levels, including proteome analysis of patient biopsies and body fluids, glioma cell lines, and animal models [6]–[9]. Despite the promise of shotgun proteomics for disease marker discovery, there has been general criticism however, that the enormous amounts of proteomic data have not yet yielded significantly novel insights into molecular mechanisms of glioma invasion and translation into either reliable biomarker candidates for diagnosis and prognosis or clinical benefit. One common pitfall of the glioma shotgun proteomic studies reviewed is the fact that whole cell lysates are examined [8]. Relatively few studies have characterized the human glioma cell surface proteome most likely attributable the difficulty and inherent challenges of plasma membrane protein analysis as well as limited amounts of human tumor samples [10]–[13]. An experimental strategy that characterizes the cell surface proteins overexpressed in GBM tumors would facilitate our understanding of the roles of these proteins in regulating the biological processes that lead to tumor invasion and would ultimately provide more reliable biomarkers and relevant molecular targets for treating gliomas.

Protein glycosylation, and in particular N-linked glycosylation, is prevalent in proteins localized on the extracellular side of the plasma membrane and in secreted proteins [14]–[16]. Importantly, several cell surface sialylated glycoproteins have been shown to be associated with the invasive potential of malignant gliomas [17]–[19]. Many clinical therapeutic targets of immuno- and ligand-directed toxins for HGG are cell surface sialylated glycoproteins (Table 2). These include interleukin 13 receptor subunit alpha 2 (IL13RA2), interleukin 4 receptor (IL4R), epidermal growth factor receptor (EGFR), transferrin receptor (TFRC), and tenascin (TNC). Therefore, it can be expected that a method for the systematic and quantitative analysis of cell surface sialoglycoproteins would be very useful for the detection of new potential diagnostic markers and therapeutic targets for malignant gliomas [20].

To detect low abundant glycosylated surface membrane proteins or peptides in complex mixtures, specific enrichment methods have to be applied, most commonly based on lectin affinity chromatography [21]–[23], hydrophilic interaction chromatography [24], [25], titanium dioxide chromatography [26], [27] or chemical linkage of the sugar moiety to surfaces [28]–[30].

Recently, a technology termed “bioorthogonal chemical reporter” (BOCR) strategy has been developed by Bertozzi et al. It enables cell surface sialoglycoprotein labeling with probes for visualization in cells and facilitates protein enrichment for proteomic analysis [31], [32]. This technology labels sialylated glycans with a specifically reactive, abiotic, azide functional group. In this two-step process, a synthetic azido sugar, the peracetylated N-azidoacetylmannosamine (Ac_4_ManNAz), is fed to cells or organisms, then enzymatically deacetylated in the cytosol and finally converted to the corresponding N-azidoacetyl sialic acid (SiaNAz), which is metabolically integrated into glycan biosynthesis to generate cell surface sialylated glycoconjugates. Once presented on the cell surface, the azide-labeled sialylated glycans can be covalently tagged with imaging probes or epitope tags with triarylphosphines via the Staudinger ligation, linear alkynes via the copper(I)-catalyzed azide-alkyne cycloaddition (abbreviated CuAAC) or cyclooctynes via the strain-promoted azide-alkyne [3+2] cycloaddition (also termed “Cu-free click chemistry”). Applications of the bioorthogonal chemical reporter strategy to noninvasive imaging and glycoproteomic analyses have been recently well documented [31]–[33].

**Figure 1:**
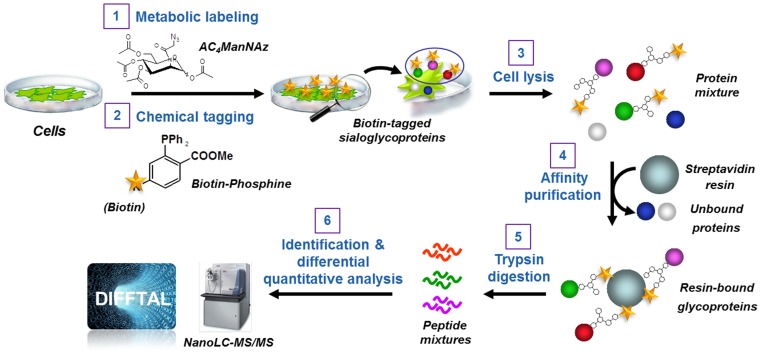
Profiling cell surface sialoglycoproteins via the bio-orthogonal chemical reporter strategy combined with quantitative shotgun proteomics. The strategy consists of six steps: 1) metabolic labeling of cells with Ac_4_ManNAz; 2) chemoselective conjugation of azide-labeled glycans with a biotin-linked phosphine; 3) cell lysis; 4) affinity enrichment of the biotin-tagged proteins on streptavidin-conjugated beads; 5) on-beads trypsin digestion of affinity-captured sialoglycoproteins; 6) identification using high-performance liquid chromatography tandem mass spectrometry (LC-MS/MS) analysis using a nano-LC-LTQ Orbitrap mass spectrometer platform and sequence database searching, and quantitative differential analysis of the sialoglycoproteins using label-free analysis with DIFFTAL (DIFferential Fourier-Transform AnaLysis) software algorithm.

In this work, proteomic profiling was performed employing BOCR technology in combination with LFQ-MS to accurately identify and quantify the individual cell surface sialoglycoproteome in human glioma tissues. We applied the strategy schematized in Fig. 1 to analyze differentially expressed cell surface sialoglycoproteins in primary cultures derived from GBM patient tumors, fetal and adult human astrocytes, as well as human NPCs. Several technical and scientific questions were addressed in this study. From the technical prospective, experiments were performed to determine if the BOCR strategy in combination with LFQ-MS provides accuracy and sensitivity enough to allow robust identification of the surface antigens or receptors already validated for the targeted therapy of HGG. More importantly, we addressed whether this technological advancement could facilitate the discovery of new tumor-associated cell surface antigens or receptors to enable better specificity for the targeting of malignant gliomas. Importantly, the effectiveness of our technical and scientific approach was validated by the identification of well-known glioma therapeutic targets and new cell surface antigens whose expression is predominantly restricted to GBM tumors which was confirmed by microarray transcription profiling, quantitative RT-PCR and immunohistochemical staining. Altogether, this result demonstrates the translational power of our quantitative sialoglyproteomics approach for the discovery of new credentialed biomarkers and therapeutic targets for treatment of malignant gliomas.

## Materials and Methods

### Ethics Statement

Inclusion of patients in this study was approved by the institutional ethical committee of the University of Rochester Medical Center (USRMC), Research Subjects Review Board (RSRB) according to their guidelines. The study complies with the Declaration of Helsinki, 1995. The subjects gave full written informed consent, and patient anonymity has been preserved. The USRMC RSRB specifically approved this study.

### Cell Culture

NPCs and astrocytes (ASG6 and ASG7 collected at 19.1 and 21.2 weeks of gestation, respectively) were isolated from human fetal brain tissues obtained at abortion as described previously [76]. Tumor cells (SIDs) were isolated from primary human GBM specimens removed from 4 male patients (53 72 years old) and maintained in short-term culture as described by Auvergne et al. [36]. The 4 GBM tumors assessed in this study could be classified according to the four GBM gene expression subclasses defined by The Cancer Genome Atlas (TCGA) (http://cancergenome.nih.gov/) as follows: proneural (SID227 and SID238), classical (SID233) and mesenchymal (SID186). Both fetal and adult samples were obtained with informed consent, using protocols approved by the University of Rochester-Strong Memorial Hospital Research Subjects Review Board (Rochester, NY, USA). Adult human astrocytes were purchased from Lonza and maintained according to the instructions provided. Human glioblastoma-astrocytoma grade III U373 MG cells obtained from the American Type Culture Collection (Manassas, VA, USA) were cultured in growth medium (MEM medium supplemented with 10% FBS, 2 mM L-glutamine, 1% MEM non-essential amino acids, 1% sodium pyruvate and ciprofloxacin) under 5% CO_2_ at 37 °C.

### Sialoglycoprotein labeling and enrichment

Cells were incubated for 2 days in culture medium containing 25 µM peracetylated N-azidoacetylmannosamine (Ac_4_ManNAz) and subsequently incubated with 50 µM biotin-phosphine reagent in Phosphate-Buffered Saline (PBS) for 1 h at room temperature. For affinity capture of biotin-tagged proteins, cell lysates were incubated for 2 h at room temperature with streptavidin beads (Thermo Fisher Scientific). After washing, bound proteins were eluted from ten percent of the beads by incubation for 15 min at 100°C with an elution buffer containing 2% (w/v) SDS, 6 M urea, 2 M thiourea, 30 mM biotin, 100 mM NaCl, 50 mM NaH_2_PO_4_ (pH 12,0). The input and flow-through fractions (5 µg proteins per sample), along with eluates, were resolved by SDS-PAGE and analyzed by Western blotting with a streptavidin-HRP conjugate (Thermo Fisher Scientific). A more detailed protocol is described in File S1.

### Flow cytometry and confocal microscopy analyses

U373 MG cells were stained with 5 µg/mL Streptavidin Alexa Fluor 488 conjugate in complete culture medium. Prior to flow cytometric analysis, cells were resuspended in 100 µl 7-amino-actinomycin D viability dye (7-AAD) at 2.5 µg/mL in PBS. The cell-associated fluorescence intensity was analyzed using a BD FACSAria Flow Cytometer (Beckton Dickinson) with linear amplification of forward and side-scatter channels. Alexa Fluor 488 and 7-AAD fluorescence signals were measured simultaneously in FL1-A and FL3-A channels, respectively. For confocal microscopy analysis, cells stained with Alexa Fluor 488 conjugate were fixed with paraformaldehyde and nuclei counterstained using the blue fluorescent DAPI dye. Confocal laser scan microscopy images were acquired with a C-Apochromat Zeiss 63 ×/1.20 W Korr UV-VIS-IR objective using a Zeiss LSM 510 Meta confocal microscope. Image acquisition and analyses were performed using Zeiss LSM 5 Pascal Confocal Microscopy Software. A more detailed protocol is described in File S1.

### Proteolytic digestion

The proteins captured on streptavidin beads were reduced in the presence of 10 mM dithiothreitol and 6 M urea in 50 mM NH_4_HCO_3_ pH 8.2 at 56°C for 30 min and then alkylated by adding 20 mM iodoacetamide for 30 min at room temperature in the dark. After the reduction and alkylation steps, beads were washed in 50 mM NH_4_HCO_3_ pH 8.2, and bound proteins were digested with trypsin (2 µg/100 µL beads 50% slurry) for 12–14 h at 37°C. After centrifugation, protein digests were collected and the beads were washed with an equal volume of 0.2% (v/v) formic acid in H_2_O. After a final centrifugation step, the supernatants were recovered, combined with the previous ones, and subjected directly to MS analysis.

### NanoLC-MS/MS analysis

Peptide digests were analyzed on a on a Ultimate/Famos/Switchos suite of instruments (Dionex) connected to a hybrid LTQ Orbitrap mass spectrometer (Thermo Fisher Scientific) with the instruments setup and parameters described in File S1.

### Proteins identification, quantification and statistical analysis

Database searches were done using an internal MASCOT server (version 2.1, matrix Science; http://www.matrixscience.com/) using the UniProtKB/Swiss-Prot protein knowledgebase (http://www.uniprot.org/) with the search parameters as described in File S1. For protein quantification, raw data were processed by an in-house label-free software, DIFFTAL (DIFferential Fourrier Transform AnaLysis) [37] as described in the in File S1. For statistical analysis, DIFFTAL data normalizations (loess normalization at sample level and median central tendency at match set level), protein ratio (Effect size) and statistic p-value (ANOVA) calculations were performed using DanteR 0.0.1 software (http://omics.pnl.gov/software/).

### Protein annotation

For subcellular localization and molecular function annotations all the proteins identified in this study were analyzed using the Ingenuity Pathway Analysis Knowledge Base (http://ingenuity.com/), Human Protein Reference Database [38], UniProtKB/Swiss-Prot protein knowledgebase, the Gene Ontology consortium (http://geneontology.org/) and the PANTHER classification system (http://www.pantherdb.org/) [39]. Transmembrane topology and signal peptide prediction of every identified protein was obtained from the UniProtKB/Swiss-Prot protein knowledgebase.

### Human gene and protein expression analyses

Gene expression data were downloaded from the BioGPS website (http://biogps.gnf.org/) [49] and from the Gene Expression Atlas database (http://www.ebi.ac.uk/gxa/). Gene expression data from ArrayExpress Archive (http://www.ebi.ac.uk/arrayexpress/) are as follow. E-MTAB-62: Human gene expression atlas of 5372 samples representing 369 different cell and tissue types, disease states and cell lines; E-MTAB-37: Transcription profiling of human multiple cancer cell lines (950 samples); E-GEOD-4290: Transcription profiling of human glioma tumor samples; E-MEXP-567: Transcription profiling of human grade II astrocytic tumor (astrocytomas) vs grade IV tumors (glioblastoma); E-GEOD-8692: Transcription profiling of brain tumor cells to assess endogenous mRNA fluctuations detects a microRNA signal in an in vivo expression set of mRNAs. Differentially-expressed genes in these microarray experiments were identified using t-statistic values. Protein expression data were downloaded from The Human Protein Atlas (HPA) portal (http://www.proteinatlas.org/) [50].

### Microarray and quantitative real-time PCR

Total RNA was isolated from cell cultures using the RNeasy Mini kit (Qiagen) according to the manufacturer’s protocol. Microarrays were performed with GeneChip HT Human Genome U133 Array Plate Set (Affymetrix) using an Affymetrix GeneChip Array Station. Plate arrays were scanned using an Affymetrix GeneChip HT Scanner. Gene expression data analysis was done with the Rosetta Resolver gene expression analysis software (version 5.0, Rosetta Biosoftware). Reverse transcription polymerase chain reaction (RT-PCR) was conducted using the High Capacity cDNA Reverse Transcription kit (Invitrogen) according the manufacturer’s protocol. Real-time PCR was performed using cDNA with TaqMan Universal PCR Master mix (Invitrogen) on an ABI Prism 7900HT Sequence Detection System (Applied Biosystems). The ID numbers for all specific primers used are listed in Table S6. Human Eukaryotic 18S rRNA Endogenous Control (Invitrogen) was used as the endogenous control for relative gene expression quantification and the comparative CT Method (ddCt) [77] was used to calculate the relative fold-difference in target gene expression between tumor cells and astrocytes. All reactions were performed in triplicate. A more detailed protocol is described in File S1.

## Results

### Generation of primary glioblastoma cell cultures

Developing targeted therapy for HGG relies largely on glioma cultures. However, it is unclear if glioma tumorigenic signaling pathways are retained under in vitro growth conditions. Several recent studies have shown that astrocytoma derived short-term cultures (less than 10 passages) do retain key aspects of the global tumor expression profile and are representative of the tumor in situ [34]–[35]. In this study, short-term cell cultures (passage <8), generated from four GBM biopsies, were grown and expanded in stem cell-permissive growth medium. Each of the resultant lines uniformly expressed the glial tumor progenitor cell marker A2B5, and each was capable of self-renewal, could be cultured as clonogenic neurospheres under serum-free conditions, and was able to initiate and propagate tumors upon xenotransplantation [36]. These results indicate that the glioma cells used in this study manifest the salient characteristics of glioma-initiating tumor progenitor cells.

### Efficient and selective labeling of cell surface sialoglycoconjugates

**Figure 2:**
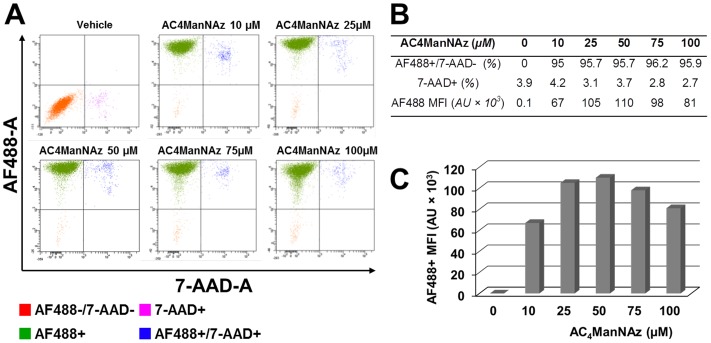
Flow cytometric analysis of azide-modified surface sialoglycoconjugates on U373 MG cells. U373 cells were incubated with increasing concentrations of Ac_4_ManNAz (10 to 100 µM) or vehicle for two days and then probed with biotin phosphine (50 µM) for 1 h at room temperature. Next, non-permeabilized cells were stained with Alexa Fluor 488 (AF488)-conjugated Streptavidin. After washing, cells were permeabilized, stained with 7-amino-actinomycin D viability dye (7-AAD), and subsequently analyzed by flow cytometry. (A) FACScan dot plots generated by the analysis of 10,000 events show Streptavidin Alexa Fluor 488 versus 7-AAD cell staining. (B) 7-AAD+: percentage of non-viable 7-AAD positive cell population; AF488+/7-AAD-: percentage of AF488 positive viable cell population; AF488 MFI: AF488 mean fluorescence signal associated with viable cell in arbitrary units (AU). (C) Dose-dependent incorporation of Ac_4_ManNAz into U373 MG cell surface sialoglycoconjugates. Data are representative of at least three independent experiments.

Sialoglycoconjugates are restricted to the extracellular compartment which includes the extracellular side of the plasma membrane, the extracellular matrix, secreted proteins, and the subcellular organelles of the secretory and endocytic pathways of cells. In order to establish that these proteins could be tagged with a molecular reporter, we conducted flow cytometry analysis on the model human astrocytoma cell line U373 MG to examine the labeling efficiency of the metabolic oligosaccharide engineering strategy (Fig. 2). Over 95% of the viable cell population was stained with streptavidin-Alexa Fluor 488 whatever the dose of azidosugar used, indicating that AC_4_ManNAz is metabolized to SiNAz homogeneously into all cells and this from the lowest dose of 10 µM. U373 MG cells treated with AC_4_ManNAz exhibited a dose-dependent increase in fluorescence that indicated the accumulation of biotin moieties on the cell surface, whereas untreated cells showed only a background level of fluorescence after exposure to the biotin-phosphine probe. The background fluorescence was identical to that observed with U373 MG cells that were not exposed to any reagents and thus represents autofluorescence of cells and not nonspecific uptake of the biotin-phosphine probe or streptavidin-Alexa Fluor 488 (data not shown). As quantified by flow cytometry, the AC_4_ManNAz–treated U373 MG cells displayed about 280–450 fold greater Alexa488-specific fluorescence intensity than the control vehicle-treated cells. Reliable fluorescence labeling was achieved at a 10 µM concentration of azidosugar, however optimal results were obtained at a concentration ranging from 25 to 50 µM. No increase in labeling was observed at concentrations higher than 50 µM. A significant decrease in labeling occurred at concentrations higher than 50 µM owing to the limited solubility of AC_4_ManNAz. At all concentrations, AC_4_ManNAz showed no cellular toxicity as assessed by morphology and 7-AAD staining. Altogether, these results demonstrate that AC_4_ManNAz was efficiently incorporated into cellular glycans with no apparent adverse effect on U373 MG cell survival.

**Figure 3:**
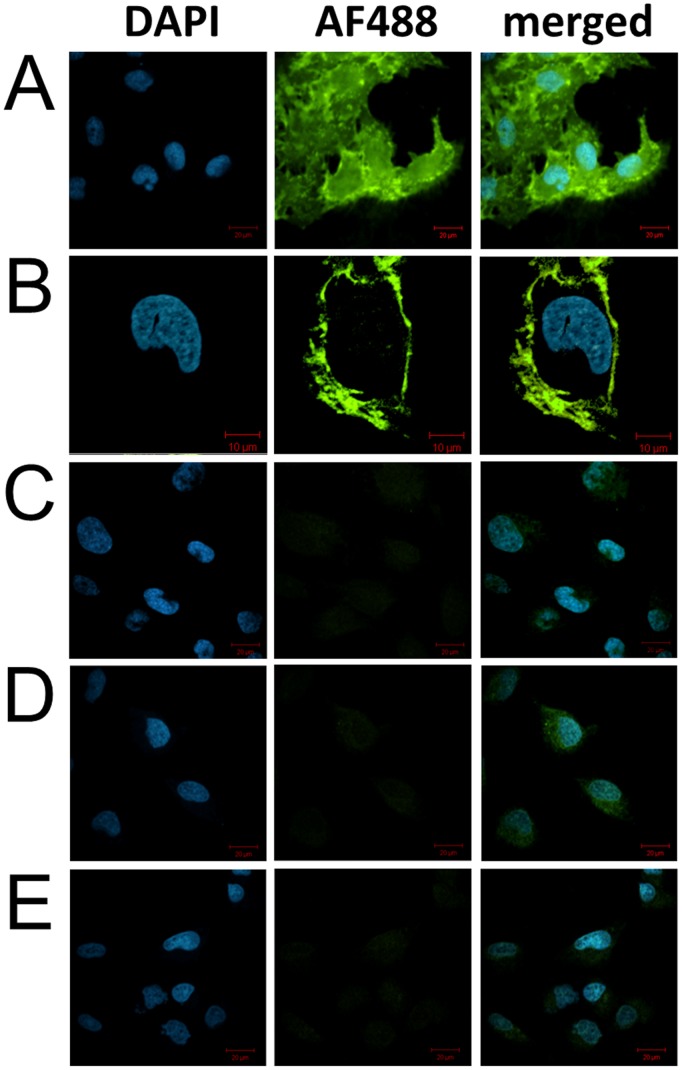
Confocal microscopy images of azide-modified surface sialoglycoconjugates on live U373 MG cells. U373 cells were incubated with 25 µM Ac_4_ManNAz (A – C) or vehicle (D, E) for two days and subjected (A, B, D) or not (C, E) to Staudinger ligation with biotin phosphine (50 µM) for 1 h at room temperature. Next, cells were stained with Streptavidin Alexa Fluor 488 at 4°C for 20 min and, after washing, fixing, and staining for the nucleus with the DAPI dye, imaged. Merged indicate that the images of cells labelled with Alexa Fluor 488 (λem = 520 nm) and DAPI (λem = 460 nm) are merged and shown in green and blue, respectively. Scale bars, 20 µm (A, C – E) and 10 µm (B).

Subsequently, confocal microscopy analysis of fluorescent streptavidin labeled cells was performed to specifically assess the cellular location of the azido-containing glycoconjugates (Fig. 3). As expected, U373 MG cells subjected to metabolic incorporation of AC_4_ManNAz followed by Staudinger ligation with biotin phosphine were intensely stained with streptavidin-Alexa Fluor 488 on the cell surface, with no apparent intracellular staining (Fig. 3A and 3B). Consistent with flow cytometric findings, staining was completely dependent on the concomitant presence of AC_4_ManNAz and Staudinger phosphine probe confirming that the labeling via azide-containing glycoconjugates is highly specific and that azide-bearing cell surface glycans were readily imaged by using streptavidin-Alexa Fluor 488 (Fig. 3C and 3D). Furthermore, there was no evidence of cell surface labeling for the control groups, confirming that background fluorescence staining is negligible (Fig. 3E). Taken together, the results suggest that AC_4_ManNAz was selectively, efficiently, and homogenously incorporated into cell surface sialoglycoconjugates.

### Affinity capture of biotinylated SiaNAz-modified proteins

**Figure 4:**
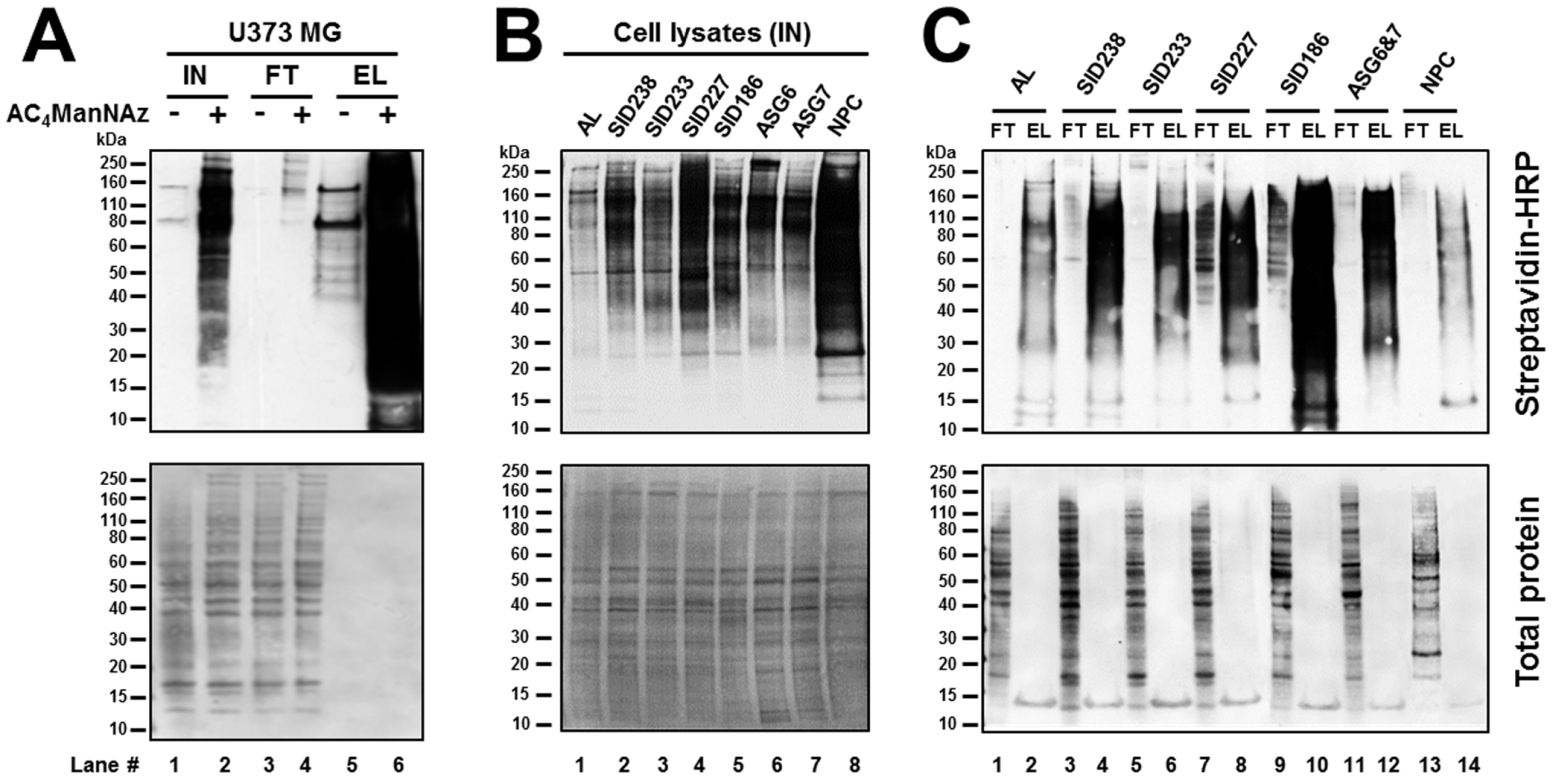
Affinity purification of biotinylated cell surface sialoglycoproteins. U373 MG cells, primary GBM cells (SIDs), adult (AL) and fetal (ASG6 and ASG7) astrocytes, and neural progenitor cells (NPC) were metabolically labelled with 25 µM Ac_4_ManNAz for 2 days and conjugated with 50 µM biotin-phosphine capture reagent. Azide-tagged and biotin-conjugated sialoglycoproteins from total cell lysate were captured by streptavidin beads, separated by SDS-PAGE and visualized using streptavidin-HRP conjugate and enhanced chemiluminescence (upper panels). Shown are the results from 5 µg of total cell lysate (Input, IN), 5 µg of the flow through material (FT) that did not bind to the beads, and 5% of the eluted material (EL) from 1 mg (SIDs and AL), 600 µg (ASG6 and ASG7 lysates combined into a single pooled ASG6&7 lysate) and 50 µg (NPC) of protein that bound to the beads. (A) Western blot analysis of U373 MG input, flow-through and eluate fractions. (B) Western blot analysis of SIDs, AL, ASG6, ASG7 and NPC input fractions. (C) Western blot analysis of SIDs, AL, NPC and ASG6&7 flow through and eluate fractions. Prior immunodetection, proteins blotted onto membranes were stained to reveal the total protein expression profile of each sample (lower panels).

Western blot analysis was performed on whole cell lysates prepared from GBM patient tumor cells, fetal and adult astrocytes, NPCs, and U373 MG cells previously labelled with AC_4_ManNAz and biotin phosphine. As shown on Fig. 4, an efficient glycoprotein labeling was observed in lysates from cells treated with AC_4_ManNAz (Fig. 4A, lane 2; Fig. 4B, lanes 1–8). With the exception of two bands at ∼80 and 150 kDa, corresponding to endogenous biotinylated proteins, no significant chemiluminescence signal was observed in lysates from cells untreated with the azidosugar (Fig. 4A, lane 1). If all human cells tested demonstrated SiaNAz-dependent glycoprotein labeling, significant variations in the labeling efficiency could be observed from one cell type to another. It is likely that the ability to metabolize AC_4_ManNAz is highly cell line-dependent. These results demonstrate that the endogenous cellular machinery can incorporate SiaNAz into proteins, which can then be selectively conjugated with biotinylated phosphine capture reagent. The resulting biotinylated, SiaNAz-modified proteins can be specifically detected using a streptavidin-HRP detection system.

The results demonstrated that proteins bearing azido sialic acid moieties are specifically detected by Western blotting and could be employed for affinity purification on streptavidin resin for their subsequent identification by mass spectrometry. Therefore, the whole cell lysates containing SiaNAz-modified proteins previously conjugated with a biotinylated phosphine capture reagent were subjected to then selectively affinity-purification using streptavidin-derivatized beads. The strong interaction between biotin and streptavidin permitted the use of harsh washing conditions to facilitate removal of any nonspecifically bound proteins. Captured proteins were eluted from the beads and subjected to SDS-PAGE and Western blot analysis using a streptavidin-HRP conjugate. In all cell types, we observed a dramatic enrichment for biotin-tagged proteins in the elution fractions from AC_4_ManNAz-treated cells (Fig. 4A, lane 6; Fig. 4C, lanes 2, 4, 6, 8, 10, 12 and 14). In contrast, only two principal chemiluminescent bands detected at ∼80 and 150 kDa, corresponding to endogenous biotin-containing proteins, were enriched in the elution fractions from untreated control cells (Fig. 4A, lane 5). Finally, little or no signal was observed in flow-through fractions containing exclusively unbound contaminants (Fig. 4C, lanes 1, 3, 5, 7, 9, 11 and 13). Taken together, these results demonstrate efficient capture and enrichment of the azide-labeled sialoglycoproteins in a single-step purification procedure.

### Selective identification of cell surface sialoglycoproteins by mass spectrometry

Having demonstrated efficient labeling and selective enrichment of cell surface sialoglycoproteins, we performed a proof-of-principle experiment to test the validity and robustness of our glycoproteomic approach. Metabolic oligosaccharide engineering was combined with LFQ-MS analysis to identify sialoglycoproteins differentially expressed between primary cultures derived from GBM patient tumors, human fetal and adult astrocytes and human NPCs, as described in detail in File S1.

**Figure 5:**
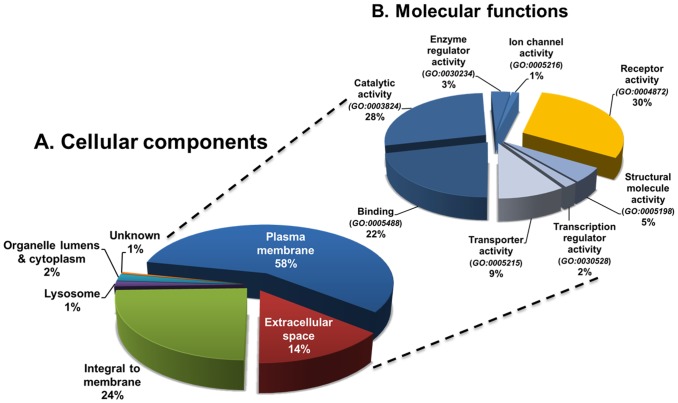
Classification of identified sialoglycoproteins by GO annotation. (A) Cellular components were assigned using Ingenuity Pathway Analysis Knowledge Base (http://ingenuity.com/), the Human Protein Reference Database (http://www.hprd.org/), and the Gene Ontology (GO) Consortium (http://geneontology.org/). (B) Molecular functions were assigned using the PANTHER classification system (http://www.pantherdb.org/). Of the 843 unique sialoglycoproteins confidently identified from the ten samples, 485 (58%) and 121 (14%) proteins could be assigned to the “plasma membrane” (GO:0005886) and “extracellular space” (GO:0005615) GO categories, respectively. A total of 819 molecular function hits were allotted to the 606 cell surface proteins. Of these, approximately one-third (34%) correspond to receptor activity including all major classes of cell surface receptor proteins.

A total of 843 unique proteins including 786 N-linked glycoproteins were confidently identified and quantified from the seven samples by DIFFTAL (DIFferential Fourier-Transform AnaLysis) software algorithm [37] and gene ontology (GO)-annotated using the Ingenuity Pathway Analysis Knowledge Base (http://ingenuity.com/), the Human Protein Reference Database (http://www.hprd.org/) [38], the Gene Ontology Consortium (http://geneontology.org/) and the UniProtKB/Swiss-Prot Protein Knowledgebase (http://www.uniprot.org/). The number of N-linked glycoproteins identified is certainly underestimated since the use of the UniProtKB/Swiss-Prot database to determine if a protein is glycosylated is notoriously under-representative. Table S1 lists the information of the 843 proteins identified from GBM tumor cells, fetal and adult astrocytes and NPCs. Of these proteins, 485 (58%) and 121 (14%) could be assigned to the “plasma membrane” and “extracellular space” GO categories, respectively, whereas 237 (28%) proteins were annotated as “intracellular” including 205 (24%) “integral to membrane” (GO:0016021) or “membrane-bounded organelle” proteins, 12 (∼1%) “lysosomal” proteins (GO:0005764) and 16 (∼2%) proteins assigned to “organelle lumens” or “cytoplasm” (Fig. 5A and Table S1). Interestingly, 601 (87%) of the 690 membrane-associated proteins and 16 (∼13%) of the 121 proteins annotated “extracellular space” contain at least one predicted TM segment and therefore are likely to be constituents of the plasma membrane. Finally, 708 (84%) of the 843 sialoglycoproteins identified in our study were predicted by the SignalP 3.0 software (http://cbs.dtu.ck/services/) to contain a signal peptide. These data suggest there was a significant enrichment of the pool of cell surface sialoglycosylated proteins using this method.

In order to classify the plasma membrane and extracellular sialoglycoproteins identified into biological context, the molecular functions of the 606 cell surface proteins were assigned using the PANTHER classification system (http://www.pantherdb.org/) [39]. A total of 819 molecular functions were assigned to these proteins and detailed in Fig. 5B and Table S2. Classification of molecular functions of the 485 plasma membrane proteins based on annotations in the UniProt Knowledgebase and the Gene Ontology Consortium revealed several classes of enriched proteins among which 156 CD-antigens (25%) and all major classes of cell surface receptor proteins. These included 32 members of the class of the G-protein-coupled receptors (GPCRs), which are known to be generally of low abundance, 32 receptor tyrosine kinases (RTKs), 2 serine/threonine receptor kinases, 10 membrane-spanning phosphotyrosine phosphatase receptors and 20 cytokine/growth factor receptors. Further 19 integrins and 72 other cell adhesion proteins that are involved in cell-cell interaction, 21 transmembrane peptidases, 77 transporters and 10 ions channels were also identified.

Significantly, the method confidently identified several cell surface glycoproteins related to malignant gliomas, previously validated as candidate molecular targets for cytotoxic and targeted agents currently examined in preclinical and clinical GBM trials (Tables 1 and 2). Among them, we found: members of the RTK superfamily involved in tumor growth, survival and angiogenesis of gliomas including epidermal growth factor receptor (EGFR), platelet-derived growth factor receptors (PDGFRs) and anaplasic lymphoma kinase (ALK). Other RTKs, such as Axl, IGF1R and EphB2/Receptor, reported to be involved in malignant glioma biology, were also identified; Integrins αυβ3and αυβ5 (ITGAV, ITGB3 and ITGB5), that are important for glioma cell migration and angiogenesis; Target proteins that were overexpressed in primary explants of human malignant gliomas relative to normal and nonneoplastic brain tissues were glycoprotein NMB (GPNMB), tenascin (TNC) and interleukin-13 receptor α2 subunit (IL13RA2). Excellent reviews describe in details all these molecular targets and associated novel agents currently in clinical development [5], [40]–[44]. CD133 (promini-1), the controversial marker of glioma stem cells [45] was also among the list in Table S1. Altogether, these results demonstrate that in terms of molecular function and abundance, a wide range of cell surface sialoglycoproteins were comprehensively identified implying that our proteomic approach is limited neither to a certain subclass of cell surface proteins nor by the dynamic range of expression. These results also highlight the potential of this surface protein profiling technology for selective identification of novel cell surface protein targets in patient cells from tumor-derived tissues. Although this strategy has been used for many years by profiling gene expression from tumor samples, mRNA expression from tumor samples often does not correlate with protein expression levels. Thus direct protein profiling offers a more reliable measure of protein expression and function.

### Accurate relative quantification of cell surface sialoglycoproteins

The object of this study was to apply our glycoproteomic approach to profile cell surface sialoglycoproteins from human GBM tissues and non-tumorous primary brain cells in order to identify tumor-specific expression of cell surface proteins as potential targets for malignant glioma targeted therapy. For biostatistics analysis of average differences in sialoglycoprotein mean intensities (“Effect sizes”), between multiple replicate samples of tumor tissues (“tumor experimental group”) and astrocytes (“astrocyte reference group”), a one-way analysis of variance (ANOVA) was performed. “Effect sizes”, a simple way of quantifying the size of the differences between the experimental and the reference group, was calculated and expressed in a Log base 2 scale (Table S1). A difference of 3 in the Log2 Effect size was considered as a significant difference.

**Figure 6:**
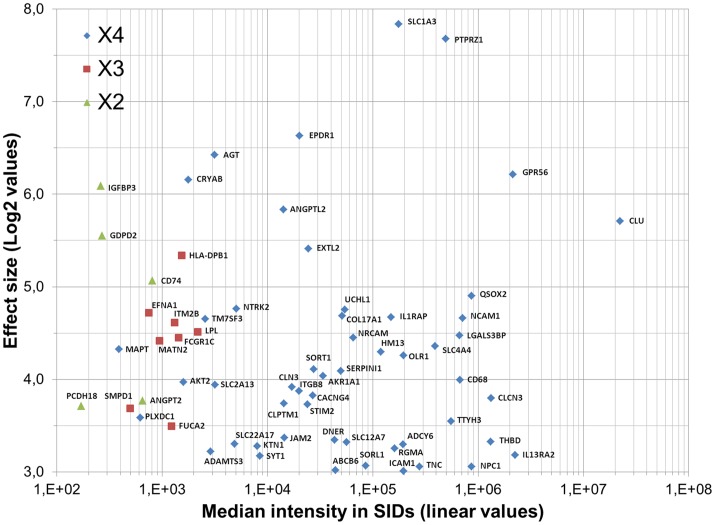
Top 65 cell surface sialoglycoproteins overexpressed in GBM patient tumors (SIDs) compared to human astrocytes. Biostatistics analysis of average differences in sialoglycoprotein mean intensities (“Effect sizes”), between multiple replicate samples of tumor tissues (“tumor experimental group”) and fetal and adult astrocytes (“astrocyte reference group”) was performed using a one-way analysis of variance (ANOVA). Effect sizes (Log2 scale) of proteins which are up-regulated (Effect size >3 and p-value <0,05) in the tumor experimental group versus the astrocyte reference group are plotted against protein median intensities (linear scale) in the tumor experimental group. The x-axis shows the median intensity values on a logarithmic scale. Among the 65 proteins up-regulated in tumors, 52 were detected in all four tumor samples (blue diamonds), while 8 and 5, were found in at least three (red squares) or two (green triangles), among the four tumor samples, respectively.

Of the 606 cell surface proteins identified and quantified with high confidence (p-value <0.05), 65 were significantly up-regulated (Effect size >3) in the tumor group compared to the astrocyte group (Table S3), of which 6 (ABCB6, AKR1A1, AKT2, CRYAB, SLC1A3 and UCHL1) were not annotated as N-glycoproteins in the UniProtKB Knowledgebase. Among the 65 proteins up-regulated in tumor cells, 51 (78%) have been described as integral or peripheral plasma membrane proteins and 14 (22%) as extracellular space proteins. Amongst suitable antigen characteristics that are critical for the identification of new glioma-specific surface antigens as putative targets for targeted toxins, abundant and homogeneous antigen expression on the external surface of all tumor cells in the majority of patients, and limited or preferably no antigen expression in vital normal tissue, are essential prerequisites. Fig. 6 shows a scatter plot of the median protein intensity in the tumor group versus the Effect size for the 65 cell surface proteins up-regulated in the tumor group. Levels of the 65 detected proteins spanned approximately five orders of magnitude. Of these, 52 (80%) were detected in all four tumor samples indicative for common expression of these proteins among GBM tumors, while 8 (12%) and 5 (8%), were found in at least three or two, among the four tumor samples, respectively. The 52 tumor-associated proteins extend over almost the entire distribution of the proteome expression. Clusterin (CLU) was the protein with the highest expression value, while protocadherin 18 (PCDH18) was the least abundant protein. Several functional protein classes were found in the PANTHER database for the 52 proteins up-regulated in the four tumor samples (Tables 3 and S3). A total of 72 molecular function hits were allotted to these proteins (Fig. S1).

**Table 3:** Top 52 cell surface sialoglycoproteins overexpressed in GBM tumor cells.

HUGO-ID	Protein expression in SIDsvs astrocytes or NPCs^a^			Gene Expression in SIDs^b^ vs astrocytes		Gene Expressionin GBM^c^	GBM tissue staining statistics (%)^d^			
	*Effect Size SIDs/Astro*	*Effect Size SIDs/NPCs*	*Intensity in SIDs*	*Array ratio*	*qPCR ratio*		*strong*	*median*	*weak*	*negative*
SLC1A3	7,84	18,22	1,77E+05	74,11	49.77	up (3)			15	85
PTPRZ1	7,68	5,50	4,91E+05	100,00	2873,52	up (2)	36	32	23	9
EPDR1	6,63	12,35	2,02E+04	ND	3,74	up (1)		55	27	18
AGT	6,42	12,19	3,15E+03	29,51	35,18	up (1)		8		92
GPR56	6,21	17,04	2,14E+06	52,87	680,07	up (3)	ND	ND	ND	ND
CRYAB	6,16	14,78	1,78E+03	2,52	283,60	up (2)	22	12	15	11
ANGPTL2	5,84	11,65	1,42E+04	2,77	251,04	up (1)			14	86
CLU	5,71	19,69	2,23E+07	9,67	22,88	up (2)		22	39	39
EXTL2	5,41	11,75	2,45E+04	−1,08		up (2)	25	58		17
QSOX2	4,90	14,98	8,67E+05	ND		down (1)	27	64	9	
NTRK2	4,76	−1,81	5,06E+03	88,10	5704,24	up/down (2/2)	54	42	4	
UCHL1	4,75	16,40	5,42E+04	1,65		up/down (2/1)	36	23	27	14
COL17A1	4,69	−3,22	5,12E+04	1,05		up (2)			30	70
IL1RAP	4,67	13,26	1,48E+05	2,72	5,46	up (4)				100
NCAM1	4,66	1,29	7,16E+05	2,97		up/down (1/2)	35	30	17	18
TM7SF3	4,65	11,99	2,56E+03	−1,07		up (1)		64	9	27
LGALS3BP	4,47	16,72	6,66E+05	2,51	1,67	up (3)	21	8	8	63
NRCAM	4,45	−0,08	6,53E+04	12,79		up (2)	10	20	40	30
SLC4A4	4,36	16,69	3,92E+05	6,21		up (2)		82	18	
MAPT	4,32	−4,12	3,91E+02	15,19		up/down (1/3)	100			
HM13	4,29	1,23	1,19E+05	ND		up (1)		17	25	58
OLR1	4,26	−0,19	1,95E+05	−7,51		up/down (2/1)	ND	ND	ND	ND
SORT1	4,11	15,07	2,75E+04	−1,08		up (3)	29	43	14	14
SERPINI1	4,09	11,05	4,98E+04	3,16		down (3)		5	32	63
AKR1A1	4,04	13,15	3,38E+04	ND		up (2)	2	5	34	39
CD68	3,99	12,69	6,75E+05	1,02	4,04	ND				100
AKT2	3,97	10,07	1,59E+03	1,10		up (1)	42	50	8	
SLC2A13	3,94	9,41	3,18E+03	ND		down (1)			9	91
CLN3	3,92	13,13	1,71E+04	−1,38		up (1)	ND	ND	ND	ND
ITGB8	3,87	5,72	2,00E+04	2,06	13,90	up (3)		4	25	71
CACNG4	3,83	11,10	2,71E+04	17,43		up (1)	ND	ND	ND	ND
CLCN3	3,80	14,15	1,33E+06	1,56	4,06	up/down (1/2)		70	30	
CLPTM1	3,74	2,40	1,43E+04	−1,28		up (1)	ND	ND	ND	ND
STIM2	3,73	0,03	2,40E+04	ND		up/down (1/1)		45	36	19
PLXDC1	3,59	9,90	6,17E+02	2,95		up (3)		18	45	37
TTYH3	3,55	13,70	5,54E+05	ND	0,88	up (2)	ND	ND	ND	ND
JAM2	3,37	3,60	1,45E+04	9,13		up (2)	ND	ND	ND	ND
DNER	3,35	11,11	4,35E+04	ND		up/down (1/1)			9	91
THBD	3,33	−0,65	1,32E+06	2,12	0,62	up/down (1/1)			4	96
SLC12A7	3,32	1,82	5,63E+04	2,92	9,82	up/down (1/1)		56	33	1
SLC22A17	3,30	−1,95	4,85E+03	−1,40		up/down (1/2)	18	73	9	
ADCY6	3,30	2,85	1,93E+05	−1,22	1,27	up (1)		9	18	73
KTN1	3,28	11,78	8,02E+03	−1,27		up/down (1)	82	18		
RGMA	3,25	1,55	1,62E+05	ND	11,15	up (1)	8	25	17	50
ADAMTS3	3,22	−0,72	2,89E+03	11,26		up (2)	ND	ND	ND	ND
IL13RA2	3,18	5,64	2,26E+06	22,12	166,33	up (3)	ND	ND	ND	ND
SYT1	3,17	1,80	8,45E+03	3,79		down (2)	8	25	58	9
SORL1	3,07	13,75	8,53E+04	15,60		down (1)		55	27	18
NPC1	3,06	16,82	8,70E+05	1,52		up/down (1/1)		64	9	27
TNC	3,06	15,87	2,79E+05	1,62	17,47	up (4)		55	18	27
ABCB6	3,02	9,78	4,43E+04	1,62		up (1)		33	25	42
ICAM1	3,01	0,21	1,96E+05	2,80		up/down (1/2)	17	11	9	63

a Proteomic analysis of differential sialoglycoprotein expression in glioblastoma multiform (GBM) cells compared to astrocytes or neural progenitor cells (NPCs). Effect size (Log2 values) indicate the standardized mean difference in protein expression level between primary tumor cells (SIDs) and astrocytes or NPCs. For each protein, the median intensity level (linear scale) in the tumor group is indicated. Normalized protein intensities used to calculate effect sizes are listed in Table S3.

b Microarray and quantitative real-time PCR (qPCR) analyses of differential gene expression in GBM cells compared to adult astrocytes. For each tumor sample, fold-change expression ratios per gene between SIDs and adult astrocytes (control) were calculated. The median fold-change expression ratio per gene is indicated. Array and qPCR data are detailed in Tables S4 and S6, respectively.

c The gene expression up- or down-regulation in glioblastoma are obtained from a large scale meta-analysis of glioblastoma public microarray data from independent studies downloaded from the Gene Expression Atlas database (http://www.hprd.org/). The number of studies the gene is over- or -under expressed in glioblastoma tissues or cell lines is indicated in parentheses. Data are detailed in Table S5.

d Protein expression profiles in malignant glioma based on immunohistochemistry for a large number of human tissues, cancers and cell lines are obtained from The Human Protein Atlas portal (http://www.proteinatlas.org/). For each indicated protein, overall malignant glioma tissue staining statistics (percentage of tumors positive for the protein studied) are reported in Table S7.

ND: not determined.

The expression status of the 52 cell surface proteins up-regulated in the tumor group compared to the astrocyte group was also evaluated in NPCs. To do so, we calculated “Effect sizes” between the “tumor experimental group” and NPCs (“NPC reference group”) using ANOVA as described previously (Table S1). Our results demonstrate clear differences in sialoglycoprotein expression patterns between the tumor group and NPCs. Of the 843 proteins confidently identified, 446 (53%) were more highly (Effect Size >3.0) expressed in the tumor group, whereas only 38 proteins (4.5%) were more highly expressed (Effect size >3.0 and p-value <0.05) in NPCs. The 35 proteins that were up-regulated in the tumor group in comparison with the astrocyte group and the NPC group are listed in Tables 3 and S3. Of these, those showing the most enriched expression (Effect size >5.0) in the tumor group include SLC1A3, PTPRZ1, EPDR1, AGT, GPR56, CRYAB, ANGPTL2, CLU and EXTL2. Importantly a number of these proteins have been previously reported to be over expressed by GBMs. Collectively these results suggest that sialoglycoprotein capture, combined with label-free LC-MS-based quantification, can reliably quantify cell surface N-linked glycoproteins in a complex sample. They demonstrated that our approach can be used to identify proven as well as new potential cell surface drug targets and/or markers directly from patient-derived GBM tissues opening the door to the potential for patient stratification and individualized treatment of the disease.

### Gene expression of glioblastoma-associated cell surface sialoglycoproteins

Our glycoproteomic results suggest increased expression of 35 cell surface proteins in GBM tumors compared to fetal and adult astrocytes and NPCs. To comprehensively validate the up-regulated proteins in the tumor group, GBM tumor cells and adult astrocytes were analyzed for gene expression by microarray analysis using The GeneChip HT Human Genome U133 Array Plate Set (Table S4). Using the fold change of the microarray gene expression ratio between tumor samples and adult astrocytes, we could evaluate gene expression for 43 out of the 52 proteins upregulated in the tumor group and listed in Tables 3 and S3. Of the 43 genes analyzed, 25 (∼58%) were up-regulated (median fold change >2) in tumors, whereas only one gene (OLR1) was more highly expressed (median fold change >2) in adult astrocytes. We compared our mRNA microarray results for these genes to those reported in a large scale meta-analysis of glioblastoma public microarray data downloaded from the Gene Expression Atlas database and found them, in general, to be in good agreement (Tables 3 and S5).

**Figure 7:**
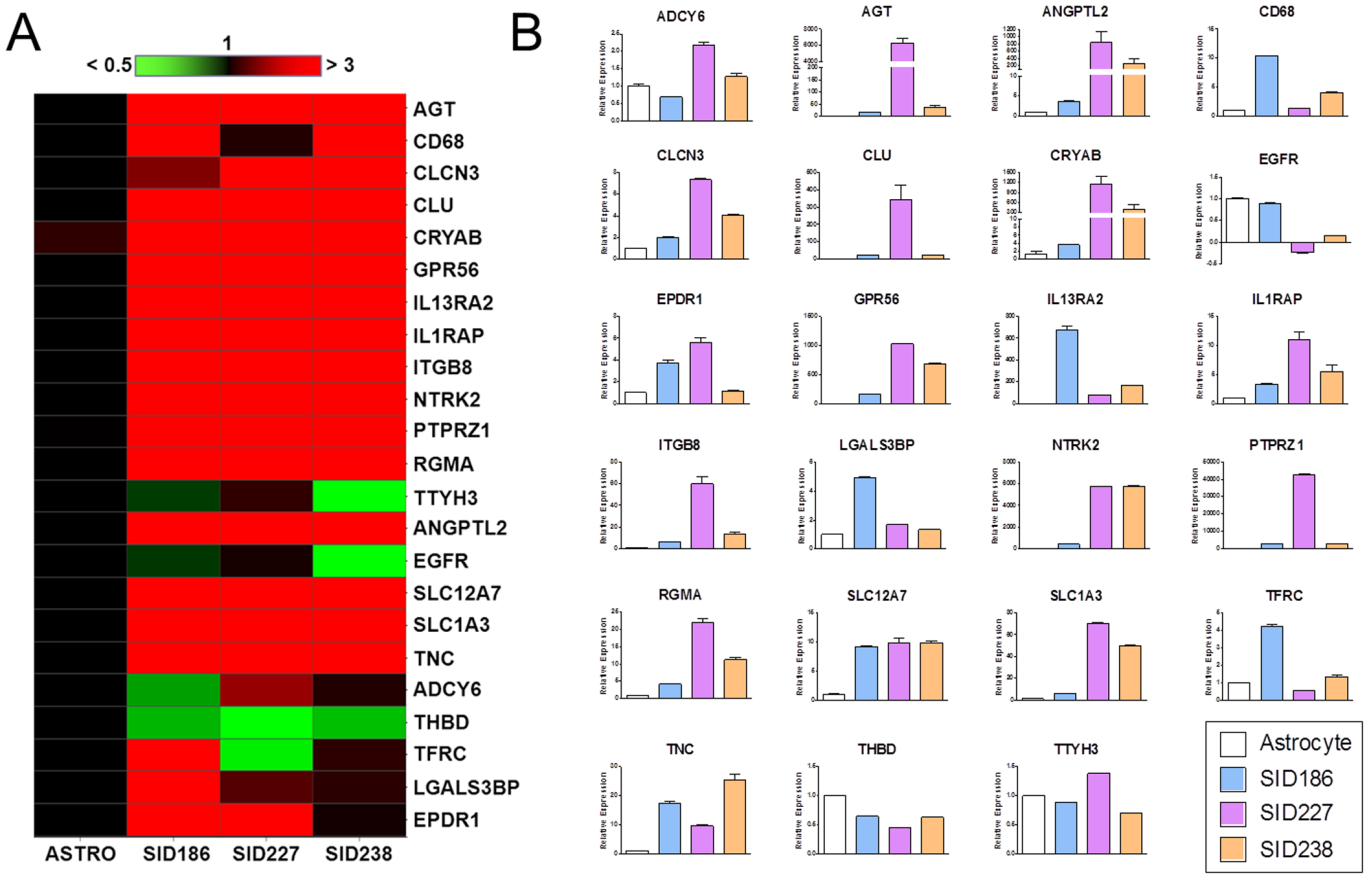
Gene expression of glioblastoma-associated cell surface sialoglycoproteins. The expression of 23 genes was analyzed by quantitative real-time PCR in GBM patient tumors and adult astrocytes, as described in the Materials and Methods section. The relative expression levels from at least triplicate experiments were averaged and expressed as means ± standard deviation (SD) (A) Heat map visualization. Bar at top gives color scale of relative expression from the mean expression: Green: Down-regulation of gene expression; Dark: No change; Red: Up-regulation of gene expression. A significant modification of gene expression was defined as a >2 down- (dark green) or >3 up-regulation (dark red). (B) Histograms represent mean expression values for each gene ± SD.

For a subset of 23 genes, the proteomic and microarray results were further validated by quantitative TaqMan real-time PCR assays for gene expression in GBM patient tumors and adult astrocytes. Of these 23 genes, 21 encode for proteins overexpressed (Effect size >3) in all four primary cultures derived from GBM patient tumors compared to human astrocytes and NPCs. The two remaining genes encode for EGFR and TFRC, two sialoglycoproteins equally expressed in the tumor group and the astrocyte group (Table S1). Expression data for individual genes are presented in Table S6. These data are also displayed in a heat map to highlight the broad similarities in gene expression that exist between tumor cells derived from 3 GBM patients relative to adult astrocytes (Fig. 7A). For 19 of the 23 selected genes, we found a significant positive correlation between mRNA expression and protein expression differences seen between GBM cells and astrocytes, indicating that a substantial proportion of protein changes between tumor cells and astrocytes were a consequence of changed mRNA levels (Table 3 and Fig. 7B). Of the 23 proteins, only four (ADCY6, LGALS3BP, THBD, and TTYH3) with upward trends had an opposite mRNA trend, these discrepancies might be the result of the post-transcription and/or post-translation regulation. Of the 23 selected genes, qRT-PCR results were consistent with the microarray results for 16 of the 19 genes whose mRNA levels were analyzed with both methods (ADCY6, AGT, ANGPTL2, CD68, CLU, CRYAB, EGFR, TFRC, GPR56, IL13RA2, ILRAP, ITGB8, NTRK2, PTPRZ1, SLC12A7 and SLC1A3). For three genes (CLCN3, THBD and TNC), microarray and qRT-PCR analyses gave inconsistent results. Overall, our results show a statistically significant correlation between protein and mRNA expression levels for the cell surface proteins up-regulated in GBM tumors. We clearly confirm overexpression of 25 cell surface proteins in GBM tumors compared to astrocytes and NPCs, as the result from microarray and qRT-PCR analyses were overall in accordance with those of our differentially expressed glycoproteomic analysis. Thus, the genes showing the highest expression levels in GBM tumors compared to astrocytes, at both mRNA and protein levels, include SLC1A3, PTPRZ1, GPR56, NTRK2, IL13RA2, NRCAM, AGT and CLU. It’s interesting to note that our proteome and transcriptome results also indicated that the expression of EGFR, the most commonly altered gene in GBM with overexpression and/or mutation occurring in at least 50% of cases [46], was not significantly different between the tumor group and the astrocyte group. Our results are consistent with previous findings showing a loss of EGFR amplification in primary cultured gliomas [34], [35], [47], [48].

### Tissue distribution of selected glioblastoma-associated sialoglycoproteins

Ideally, an optimal cell surface target for immunotoxins or ligand-directed toxins should be overexpressed or uniquely expressed on human tumor cells at a significantly high level with limited or preferably no target expression on vital normal tissue. To study human tissue distribution of the 52 selected cell surface proteins overexpressed in human GBM tumors compared to non-tumorous brain cells, we queried different human gene and protein expression web-accessible databases. BioGPS gene portal (http://biogps.gnf.org/) [49] provides information on gene expression patterns from a diverse set of tissues and cell types. The Human Protein Atlas (HPA) portal (http://www.proteinatlas.org/) [50] is a database describing protein expression profiles based on immunohistochemistry for a large number of human tissues, cancers and cell lines. The distribution pattern and relative abundance of selected cell surface proteins and corresponding mRNAs in human normal and GBM tissues are shown in Table S7. Among the 52 glioblastoma-specific proteins, the majority of them displayed moderate to strong expression in several critical (vital) normal tissues. Those weakly expressed or barely detected in most human normal tissues include ANGPTL2, ICAM1, IL1RAP, IL13RA2, ITGB8, LGALS3BP, PTPRZ1, SLC1A3, THBD and TNC. Several proteins are exclusively or predominantly expressed in the brain. SERPINI1 and DNER are selectively expressed at high levels in neuronal and/or Purkinje cells. PTPRZ1 is highly expressed in the brain with highest levels in neuropil. SLC1A3 is expressed at a low level in neuropil and subsets of glial cells. THBD and IL13RA2 are only weakly expressed in placenta and testis, respectively, and suggest that these may be targets for future therapeutics. HPA illustrates moderate to strong positive immunostaining in malignant gliomas for most of the 52 selected cell surface proteins, corroborating well our glycoproteomic data. Among the most highly up-regulated proteins in glioblastomas, we find AKT2, KTN1, MAPT, NCAM1, NTRK2, PTPRZ1, QSOX2, SORT1 and UCHL1 (Tables 3 and S7). To note that AKT2, MAPT (also called Tau) and UCHL1 are intracellular proteins which have never been described to be N-glycosylated. However, AKT2 [51] and MAPT [52] can translocate from the cytosol to the plasma membrane upon a phosphorylation-dependent and dynamic process and UCHL1 can be secreted from neurons by an unconventional pathway [53]. Our study does not assert that these proteins are indeed N-glycosylated or have been co-isolated with a sialylated component.

## Discussion

Current therapy for glioblastoma consists of surgical resection followed by radiation and chemotherapy. This therapeutic strategy is limited by toxicity to systemic tissues and surrounding brain. Despite aggressive therapy, the prognosis for patients with high-grade astrocytic tumor remains dismal. The highly heterogeneous and diffuse nature of astrocytic tumors calls for the development of novel therapies for this deadly disease. Over the past two decades, a novel class of investigative drug candidates for the treatment of malignant brain tumors has emerged: recombinant fusion protein conjugates armed with cytotoxic agents targeting tumor-specific cell surface antigens. The clinical applicability of these cytotoxins as a safe and viable therapy for malignant gliomas is currently being investigated. Thus far, results from several clinical trials are encouraging, as disease stabilization and patient survival prolongation have been observed in multiple cases. Further progress and improved clinical response will depend on the identification of new antigenic targets on tumor cells and the development of new cytotoxins targeting different tumor antigens which, administered in combination, should be able to kill cancer cells more effectively while leaving healthy cells unharmed. Therefore, developing new experimental approaches to selectively interrogate cell surface proteins should facilitate the discovery of new biomarkers for HGG diagnosis, prognosis, and help define new therapeutic targets.

Over the past decade, several reports have emerged reporting the analysis of the whole glioma proteome using cells lines, animal models, as well as human tissues or bodily fluids. A comprehensive overview of these studies was recently published [8], [9]. However, very few studies have focused on the comprehensive profiling of the cell surface proteome of human glioma cells.

Applying isobaric peptide tagging chemistry (iTRAQ) combined with two-dimensional LC and MALDI-TOF/TOF mass spectrometry, Rajcevic et al. quantified 1,460 proteins in membrane-enriched protein fractions of serially transplanted glioma xenografts in rat [10]. More recently, Polisetty et al. quantified 1,199 membrane proteins from the microsomal fraction of clinical specimens of GBM using high-resolution LC-MS/MS mass spectrometry and quantitation by iTRAQ [12]. The limitations of these approaches, however, are difficulty to obtain homogenous and highly enriched plasma membrane protein isolates, potential inefficient chemical labelling, sample loss due to additional purification steps, and chemical side reactions. Multi-lectin affinity chromatography coupled with mass spectrometry based label-free quantitative proteomics methods have been applied by He et al. to quantify over a hundred N-glycoproteins from glioblastoma cancer stem cells [11]. The major drawback of the lectin capture method is the relatively weak binding of lectins to oligosaccharides, which may introduce large amounts of non-specific adsorption of abundant proteins. Although all of these methods were able to identify membrane proteins to some extent, they still lack the specificity and selectivity required for a conclusive and comprehensive analysis of the surface membrane proteome. By using the chemoproteomic cell surface capture technology in combination with MS technology, Bock et al. identified 633 cell surface N-glycoproteins from primary and established human GBM cell lines. Although this extensive GBM surfaceome analysis definitively provided unprecedented detailed information about the quality of cell surface exposed N-glycoproteins, it only enabled an approximate estimation of their abundance in tumor cells [13].

In the present study, we applied the BOCR strategy in combination with LFQ-MS to identify cell surface N-linked sialylated glycoproteins that are differentially expressed in several human primary HGGs in short-term culture compared with fetal and adult astrocytes and NPCs isolated from human brain. Low passage (<8) primary GBM lines derived from resected patient tumors shared many of the characteristics of glioma stem cells such as self-renewal potential, neurosphere formation and tumorigenicity. Therefore they represent an appropriate model for the identification and evaluation of novel therapeutic targets for HGG. Applications of the BOCR strategy to noninvasive imaging and glycoproteomic analyses have been the subject of numerous reviews [31]–[33]. However, to our knowledge, our data is the first to demonstrate its application in proteomics-based discovery of novel biomarkers and therapeutic targets in human gliomas. The technology developed involves metabolic labeling of sialylated glycans with AC_4_ManNAz, a synthetic azido sugar. Then, azide-labeled sialylated glycans can be covalently tagged with a biotinylated triarylphosphine probe via the Staudinger ligation, a highly specific bioorthogonal reaction between an azide and a phosphine. Our flow cytometry results and confocal images demonstrated that AC_4_ManNAz was selectively, efficiently, and homogenously incorporated into cell surface sialoglycoconjugates. They confirmed the high selectivity and specificity of the Staudinger ligation for labeling cell surface azide-labeled sialoglycoconjugates in astrocytes, NPCs, and glioma cells with no apparent adverse effect on survival. We further demonstrated that Staudinger ligation of a biotin phosphine tag allowed for selective and efficient isolation of azide-labeled sialylated glycoproteins in a single-step purification procedure. LTQ-Orbitrap MS analysis of the targeted proteins identified with at least two peptides/protein and a high annotation confidence (≥95%) a total of 843 unique quantifiable proteins from the seven samples among which 121 (14%) and 485 (58%) proteins being secreted or plasma membrane proteins, respectively. These cell-surface proteins include 156 CD-antigens, all major classes of cell surface receptor proteins, transporters, and adhesion proteins. In total, 801 proteins were annotated as glycoproteins using the UniProtKB Knowledgebase including 786 N-glycoproteins. Among the 42 non-glycosylated proteins, 11 were predicted to contain a signal peptide (SignalP) and sites of N-linked glycosylation (NetNGlyc) (data not shown). These results showed a high degree of specificity for the detection of cell surface proteins, with less than 2% of identified proteins resulting from co-isolation of intracellular and non-glycosylated proteins. To the best of our knowledge, this represent the most extensive data set of cell surface N-glycoproteins confidently identified so far in GBM tissues. The proteins identified in human GBM tissues and non-tumorous brain cells were quantified using a label-free analysis with DIFFTAL software algorithm. DIFFTAL is an automated tool for label-free quantitation analysis using an efficient peptide alignment approach, spectral data validation and extracted ion current (XIC) as quantitative index [37]. XIC of the peptide is the intensity of corresponding ions in MS survey scans. Out of the 606 cell surface proteins identified and quantified with high confidence, we found 35 proteins significantly up-regulated (Effect size >3) in the tumor group compared to the astrocyte group and NPCs in all four primary cultures derived from GBM patients indicating a common expression of these proteins among HGGs. For 25 proteins, quantitative proteomic results were validated by gene expression analysis using microarrays and/or qRT-PCR. Furthermore, our results proved to be in good agreement with those reported in a large scale meta-analysis of glioblastoma public microarray data downloaded from the Gene Expression Atlas database.

Among the sialoglycoproteins overexpressed in GBM cells identified in this study, we found well-known proteins reported to be involved in various aspects of glioma biology (e.g., PTPRZ1, GPR56, TNC, IL13RA2, ICAM1, NCAM1, THBD and NTRK2) as well as some specific glioma-associated cell surface antigens or receptors that are targets for cytotoxic and targeted agents currently under clinical trials for glioblastoma (PTPRZ1, IL13RA2, TNC and TFRC) [5]. Our “re-discovery” of proteins previously validated as glioma biomarkers/therapeutic targets provides a proof-of-principle for our methodology. In addition to these well-established glioma markers, we identified several other proteins that have never been associated with glioma biology including (e.g., SLC1A3, CLU, LGALS3BP, ANGPTL2, CRYAB and ITGB8). Among the most abundant sialoglycoproteins (median intensity >150,000) over-expressed in tumor cells, those with the largest Effect size (Effect size >5.0) include SLC1A3, PTPRZ1, GPR56 and CLU. GPR56 is specifically overexpressed in human astrocytic tumors and functions in tumor cell adhesion and invasion [54]. PTPRZ1 is one of the most highly expressed receptor tyrosine phosphatases in the brain. In the adult human brain, PTPRZ1 is selectively expressed by glial progenitor cells of which it regulates their self-renewal and fate [55], [56]. The role of PTPRZ1 in glioma tumorigenesis has been extensively studied [57]. Inhibition of PTPRZ1 expression with small interfering RNA suppresses glioblastoma growth in vitro and in vivo [58]. Finally, in vivo studies showed that an anti-PTPRZ1 immunotoxin (7E4B11-SAP) could significantly delay human U87 glioma tumor growth in a mouse xenograft model [59]. Clusterin (CLU), a pleiotropic protein with a broad range of functions, has recently drawn much attention because of its association with cancer promotion and metastasis. It is involved in prosurvival and apoptosis processes that are carried out by two different forms. Secreted clusterin isoform (sCLU) is cytoprotective and its prosurvival function is the basis of the current phase I/II clinical trials against prostate, lung and breast [60], [61]. In colorectal cancer (CRC), the expression of sCLU isoform directly correlates with tumor aggressiveness and with the metastatic potential of the tumor. The progressive increase of the sCLU in colorectal tumors correlate to a significant increase of CLU in serum and stool of CRC patients, suggesting that sCLU could represent a diagnostic molecular marker for colon cancer screening [62], [63]. The other 15 differentially expressed glycoproteins displaying a significant increase in the tumors compared to astrocytes include Tenascin-C (TNC) and Interleukin-13 receptor subunit alpha-2 (IL13RA2), two cell surface sialoglycoproteins currently used preclinically and clinically as targets for a variety of therapeutic approaches using targeted cytotoxins [64], [65]. LGALS3BP, also known as 90K, is a highly glycosylated, secreted protein extensively studied in human cancer. High expression levels of 90K are associated with a shorter survival, the occurrence of metastasis or a reduced response to chemotherapy in patients with different types of malignancy [66]. Other proteins overexpressed in astrocytomas have also been implicated in tumor growth and invasion, such as the inter-cellular adhesion molecule 1 (ICAM-1) [67], the neural cell adhesion molecule NCAM1 [68] and the thrombomodulin (THBD) [69]. The polysialylated form of the cell surface glycoprotein neural cell adhesion molecule (PSA-NCAM) is overexpressed in human GBM and is considered as an adverse prognostic factor for GBM patients [70]. Notwithstanding the universal expression of NCAM1 by neuronal cells, three clinical studies have used anti-NCAM1 antibodies in patients. mAb UJ13A was shown to accumulate in gliomas by virtue of disruption of the blood brain barrier locally [71], and another antibody, ERIC-1 armed with radionuclides iodine-131 or yttrium-90, was used in a therapeutic setting in resected glioma cavities with some clinical benefit [72], [73]. Among the lowest abundant proteins (median intensity <10,000), those showing the most enriched expression (Effect size >5.0) in the tumor group compared to the astrocyte group include NTRK2 (also known as TrkB), a tyrosine-protein kinase receptor of the Trk family involved in the development and/or maintenance of the nervous system. Several studies report the expression of TrkB in adult human astrocytes and astrocytic gliomas and suggest a role of this receptor in tumor pathogenesis, especially in the early stage [74], [75].

Ideally, an optimal cell surface target for immunotoxins or ligand-directed toxins should be overexpressed or uniquely expressed on human tumor cells at a significantly high level with little or no expression in normal vital tissues. To evaluate human tissue distribution of the 52 selected cell surface sialoglycoproteins overexpressed in human GBM tumors compared to non-tumorous brain cells, two complementary methods were employed. We examined protein expression patterns in normal human tissues, cancer and cell lines using high quality immunohistochemistry data available at the HPA web portal. We also examined the expression status of the corresponding transcripts in normal human tissues and cells using microarray datasets available at the BioGPS Gene Portal web site. Our comprehensive gene and protein expression profiles analyses revealed that the majority of glioblastoma-specific proteins displayed moderate to strong expression in several vital normal tissues. Among those weakly expressed or barely detected in most human normal tissues and cells, we found well-known GBM targets (IL13RA2, PTPRZ1 and TNC) as well as new integral plasma membrane glycoproteins that were not reported by previous proteomic or transcriptomic analyses (ANGPTL2, ICAM1, IL1RAP, ITGB8, LGALS3BP, SLC1A3 and THBD). These may be important targets for future evaluation of glioma therapies.

The aim of the current study was to build a high confidence, tumor-associated, differentially expressed cell surface glycoprotein panel from clinical specimens of GBM, as a first step toward their targeted validation in a clinical setting. For this, we analyzed differentially expressed cell surface sialoglycoproteins from low passage primary human GBM cells derived from resected patient tumors in comparison to adult and fetal astrocytes as well as neural progenitor cells using a BOCR strategy in combination with LFQ-MS. Glycoproteins identified include several key members implicated in GBMs in earlier reports as well as new cell surface proteins whose expression is predominantly restricted to human GBM tumors. The described protocol provides an essential framework for identifying and evaluating these cell surface antigens as targets for localizing or cytotoxic antibody- or ligand-directed conjugates and as phenotypic markers for classifying glial tumors according to their lineage or stage of differentiation. The results also demonstrate that this method can identify new therapeutic oncology targets directly from primary patient tumors using an unbiased strategy where immunoassay reagents are not available. There is also the promise to proteomically profile individual patient tumors to specifically identify personalized therapeutic strategies to eliminate their tumor burden. While the results of this pilot study are very encouraging, they must however be statistically validated in a large-scale study on multiple clinical samples. Altogether, the results demonstrated the power of our quantitative sialoglyproteomic approach for the discovery of new biomarkers and therapeutic targets for malignant gliomas.

## Supporting Information

Figure S1 **PANTHER classification of top 52 surface sialoglycoproteins overexpressed in GBM tumor cells compared to astrocytes.** Molecular functions were assigned using the PANTHER classification system (http://www.pantherdb.org/). A total of 72 molecular function hits were allotted to these proteins and classified as detailed in Table S2.
(TIF)

Table S1 **Sialoglycoproteins identified and quantified in GBM cells, astrocytes and neural progenitor cells.**
(XLSX)

Table S2 **PANTHER classifications of cell surface sialoglycoproteins identified in glioblastoma cells, astrocytes and neural progenitor cells.**
(XLSX)

Table S3 **Top 65 cell surface sialoglycoproteins overexpressed in GBM patient tumor cells.**
(XLSX)

Table S4 **Microarray analysis of gene expression in GBM tumor cells and adult astrocytes.**
(XLSX)

Table S5 **Microarray gene expression analysis of the top 52 sialoglycoproteins over-expressed in GBM tumor cells.**
(XLSX)

Table S6 **Real-time PCR gene expression analysis of GBM-associated sialoglycoproteins.**
(XLSX)

Table S7 **Human tissue distribution of the top 52 selected sialoglycoproteins overexpressed in GBM tumor cells.**
(XLSX)

File S1 **Supporting Materials and Methods.**
(DOCX)
