# Supplementary figures and images for: Identification of Novel Tumor-Associated Cell Surface Sialoglycoproteins in Human Glioblastoma Tumors Using Quantitative Proteomics
Source: PLoS One. 2014 Oct 31;9(10):e110316. doi: 10.1371/journal.pone.0110316 (PMC4216004; doi:10.1371/journal.pone.0110316)

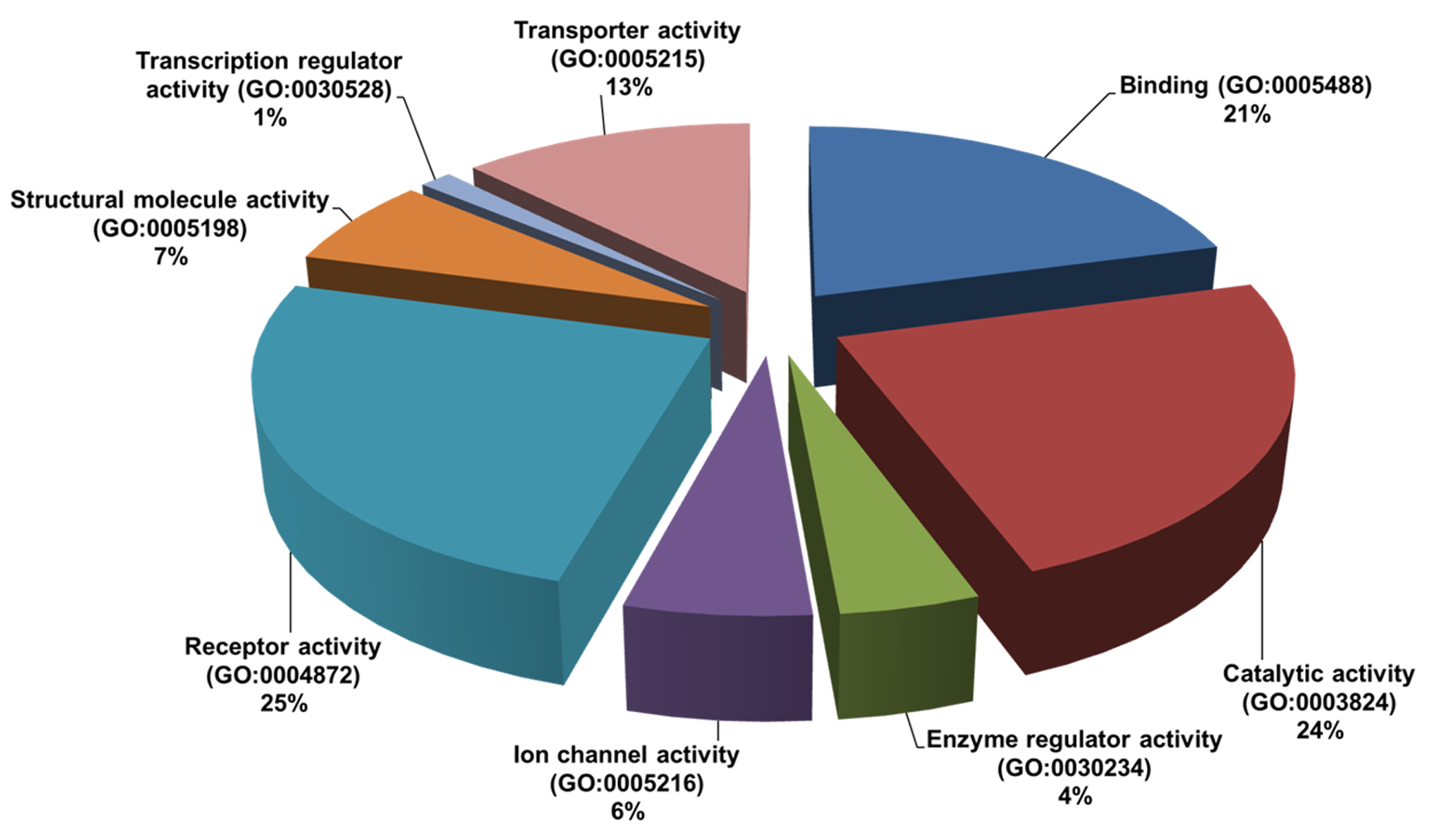

Supplement: Figure S1 — PANTHER classification of top 52 surface sialoglycoproteins overexpressed in GBM tumor cells compared to astrocytes. Molecular functions were assigned using the PANTHER classification system (http://www.pantherdb.org/). A total of 72 molecular function hits were allotted to these proteins and classified as detailed in Table S2. (TIF) [file pone.0110316.s001.tif]
