# Supplementary material for: Identification of Novel Tumor-Associated Cell Surface Sialoglycoproteins in Human Glioblastoma Tumors Using Quantitative Proteomics
Source: PLoS One. 2014 Oct 31;9(10):e110316. doi: 10.1371/journal.pone.0110316 (PMC4216004; doi:10.1371/journal.pone.0110316)
Supplement: File S1 — Supporting Materials and Methods. (DOCX) [file pone.0110316.s009.docx]

**SUPPORTING MATERIALS AND METHODS**

***Materials***

Fetal bovine serum (FBS), ciprofloxacin and all chemicals were obtained from Sigma Aldrich unless noted otherwise. High capacity streptavidin agarose resin, streptavidin horseradish peroxidase (HRP) conjugate and Pierce BCA protein assay kit were purchased from Thermo Fisher Scientific. Phosphate-buffered saline, Gibco^®^ Minimum Essential Media (MEM), Streptavidin Alexa Fluor^®^ 488 conjugate, Gibco^®^ Hanks Balanced Salt Solution (HBSS) blue fluorescent DAPI dye, NuPAGE® Novex^®^ 4 – 12% Bis-Tris gels, NuPAGE^®^ MES SDS Running Buffer and Human Eukaryotic 18S rRNA Endogenous Control were purchased from Invitrogen. Hybond-C Extra nitrocellulose membranes, ECL™ Western Blotting Detection Reagents, and Hyperfilm ECL were obtained from GE Healthcare. XT sample buffer (4×) was from Bio Rad Laboratories. Blot-FastStainTM Kit was obtained from Millipore. 7-amino-actinomycin D viability dye (7-AAD) was purchased from BD Pharmingen, and Sequencing Grade Modified Trypsin was from Promega. Peracetylated N-azidoacetylmannosamine (Ac4ManNAz) and biotin phosphine were synthetized as described by Laughlin et al. (*98*).

***Sialoglycoprotein Labeling and Enrichment***

U373 MG cells (5 × 10^6^ cells), human glioma primary cells and astrocytes (5 × 10^6^ cells), and human neural progenitor cells (1 × 10^6^ cells) were incubated for 2 days in culture medium containing 25 µM peracetylated N-azidoacetylmannosamine (Ac4ManNAz), leading to the metabolic incorporation of the corresponding N-azidoacetyl sialic acid (SiNAz) into their surface N-linked glycoproteome. Cells were washed twice with Phosphate-Buffered Saline (PBS, 0.01 M phosphate, 0.15 M NaCl, pH 7.4) and incubated with 50 μM biotin-phosphine reagent in the same buffer for 1 h at room temperature. After washing twice with PBS, cells were scraped off the dishes and lysed in 1% (w/v) SDS in PBS (lysis buffer). The cell lysates were clarified by centrifugation at 22,000 × g for 10 min and the total protein content of the clear supernatants (input fractions, IN) was assessed using the Thermo Scientific Pierce BCA Protein Assay. For affinity capture of biotin-tagged proteins, tumor and adult astrocyte lysates (1 mg proteins in 5 mL of 0.2% (w/v) SDS in PBS) were incubated for 2 h at room temperature with a 50-µL aliquot (equal to 100 μL of a 50% slurry) of streptavidin beads that have been prewashed three times with PBS. Due to the scarce total protein amount recovered in fetal astrocyte cell lysates, 400 µg for ASG6 and 200 µg for ASG7, both lysates were combined (600 µg proteins in 3 mL of 0.2% (w/v) SDS in PBS) prior to incubation with 30 µL streptavidin beads (50% slurry). Due to the low number of neural progenitors available for the study, the total protein amount recovered in whole cell lysates was also very low. Only a small quantity of the neural progenitor cell lysate (50 µg proteins in 2 mL of 0.2% (w/v) SDS in PBS) was incubated with 60 µL streptavidin beads (50% slurry) as described above. After binding, beads were pelleted by centrifugation and the flow-through fractions (FT) containing unbound proteins were saved for further analysis. The beads were successively washed with 0.2 % (w/v) SDS in PBS, 3 M NaCl, and PBS. After washing, bound proteins were eluted from ten percent of the beads (eluates, EL) by incubation for 15 min at 100°C with an elution buffer containing 2% (w/v) SDS, 6 M urea, 2 M thiourea, 30 mM biotin, 100 mM NaCl, 50 mM NaH_2_PO_4_ (pH 12,0). The input and flow-through fractions (5 µg proteins per sample), along with eluates (whole fractions), were completed with XT sample buffer containing 10% (v/v) 2-mercaptoethanol and heated at 60°C for 30 min before Western blot analysis. Protein samples (IN, FT and EL) were resolved by SDS-PAGE on NuPAGE^®^ Novex^®^ 4 – 12% Bis-Tris gels using the NuPAGE^®^ MES SDS Running Buffer according to the manufacturer’s instructions. Proteins from SDS-PAGE were transferred to nitrocellulose membranes and stained with the Blot-FastStainTM Kit to determine transfer efficiency and reveal the protein expression profile of each sample. Blots were probed with streptavidin-HRP conjugate. Immune complexes were visualized with enhanced chemiluminescence using the Amersham ECL Western Blotting Detection Reagents (GE Healthcare) and X-ray films. Autoradiographs were scanned using the GS-800 Calibrated Densitometer (Bio-Rad Laboratories).

***NanoLC-MS/MS analysis***

LC-MS/MS experiments were performed on an Ultimate/Famos/Switchos suite of instruments (Dionex) connected to a hybrid LTQ Orbitrap mass spectrometer (Thermo Fisher Scientific) equipped with a nanoelectrospray source. Tryptic digests were loaded onto a trap column (100 Å C18 Pepmap, Dionex, 5 mm × 300 µm,) and washed with 0.2% formic acid at 30 µL/min using the Switchos pump for 5 min. Peptides were then eluted on a C18 reverse-phase nanoflow column (100 Å C18 Pepmap, Dionex, 150 mm × 75 µm) with a linear gradient of 5 – 40% solvent B (H_2_O/CH_3_CN/HCOOH, 10:90:0.2, by vol.) for 125 min, 40-90% solvent B for 20 min, and 90% solvent B for 5 min, at a flow rate of 200 nL/min. The mass spectrometer was operated in the data-dependent mode to automatically switch between MS and MS/MS acquisition. Survey full scan MS spectra (from m/z 300 – 1700) were acquired in the Orbitrap with a resolution of 60,000 at m/z 400. The AGC was set to 1 × 10^6^ with a maximum injection time of 500 ms. The most intense ions (up to 5) were then isolated for fragmentation in the LTQ linear ion trap using a normalized collision energy of 35% at the default activation q of 0.25 with an AGC settings of 1 × 10^5^ and a maximum injection time of 100 ms. The dynamic exclusion time window was set to 900 s. Samples were injected in triplicate. All m/z selected for MS/MS during the first LC-MS/MS experiment were excluded of MS/MS process for the next LC-MS/MS run (generation of a reject mass list with a 10 ppm m/z window and a 10 min retention time window, see LC-MS/MS data processing). The third run was then performed with a reject mass list generated from the 1st and 2nd LC-MS/MS experiments. Ion selection threshold was set to 80,000 counts for the 1st LC-MS/MS run and to 40,000 and 20,000 for the 2^nd^ and 3^rd^ LC-MS/MS experiments, respectively.

***LC-MS/MS data processing***

LC-MS/MS data, acquired using the Xcalibur software (version 2.07, Thermo-Fisher Scientific), were processed using a home-made Visual Basic program software developed using XRawfile libraries distributed by Thermo-Fisher Scientific. This program generates 4 different files. The first one corresponds to a MS/MS peak list (MGF file) which is used for database searching. The MGF file contains the exact parent mass and the retention time (RT) associated with each LTQ-MS/MS spectrum. The exact parent mass is the ^12^C isotope ion mass of the most intense isotopic pattern detected on the high resolution Orbitrap MS parallel scan and included in the LTQ-MS/MS selection window. The RT is issued from the LTQ-MS/MS scan. The second file is a MS/MS log file which reports, for each acquired MS/MS, the scan number, the ^12^C isotope exact mass, the RT and the parent filter (LTQ selection window). The third file corresponds to the conversion of the high resolution MS raw data file into a “csv” format file which will be used for quantitative analysis. The last file is an exclude list text file which contains the ^12^C isotope precursor ion mass with the corresponding start/end exclude RT 10 min window. The Xcalibur software uses this exclude list as the reject mass list (specifies which parent ions cannot trigger a dependent scan) during the 2^nd^ and 3^rd^ LC-MS/MS experiments (see nanoLC-MS/MS analysis).

***Database searching***

Database searches were done using our internal MASCOT server (version 2.1, matrix Science; http://www.matrixscience.com/) using the Swiss-Prot human database containing 534,242 entries. The search parameters used for post-translational modifications were a fixed modification of +57.02146 Da on cysteine residues (carboxyamidomethylation) and dynamic modifications of +15.99491 on methionine residues (oxidation), of +42.010565 on protein N-terminal residues (N-terminal acetylation) and -17.026549 on N-terminal glutamine residues (N-Pyroglu). The precursor mass tolerance was set to 5 ppm and the fragment ion tolerance was set to 0.5 Da. The number of missed cleavage sites for trypsin was set to 3. Mascot result files (“.dat” files) were imported into Scaffold software (http://www.proteomesoftware.com/). Queries were also used for XTandem parallel Database Search. The compiled results of both database searches were exported.

***Quantitative analysis***

Relative quantification was performed using in-house software, DIFFTAL (DIFferential Fourrier Transform AnaLysis) (*37*). DIFFTAL is a set of software tools developed in Sanofi under MatLab environment (http://www.mathworks.com/) for label-free differential analysis of complex proteomic mixture dedicated to LTQ/Orbitrap data. DIFFTAL runs in 3 main steps: (1) Feature detection, (2) MS matching and (3) MS/MS matching.

*Step1, Feature detection*. Each LC/MS file is treated independently for feature detection. The signal apparition is detected scan by scan by analyzing the evolution of the average signal of 3 consecutive scans. Feature detection is achieved using the peptide isotopic patterns calculated with “Averagine” algorithm (*99*). In case of overlapped peptide signals, the solution is a linear combination of theoretical patterns which minimizes the distance with the detected signal. At the end of the process, a matrix of the features detected in the 3D space (m/z, RT and intensity) is stored. This matrix contains links to retrieve the corresponding processed signals, which are stored in a temporary data bank.

*Step2, MS matching*. All LC-MS data are matched together using a progressive alignment procedure. The most intense detected features are first matched in agreement with m/z and RT precision windows defined by the user. All peptides are then used to compute a specific RT alignment model. A definitive RT window is calculated according to the dispersion observed between real and calculated RTs. Finally, every remaining unmatched m/z is checked by going back to the processed signal stored during the feature detection step.

*Step3, MS/MS matching*. MS/MS Spectrum reports exported from Scaffold are matched with the matrix of detected features using the corresponding acquisition MS/MS log files (see LC-MS/MS data processing). This matching requires starting and ending time points of each feature. Indeed, the RT feature is the time at the maximum intensity of the observed MS signal, whereas the MS/MS spectrum is recorded at any time during the peptide elution. In case of ambiguity, the comparison between the exact isotopic profile calculated from the MS/MS sequence and the detected signal at the feature RT is used for sorting. Because about 5 – 10% of the identified peptides are lost during the MS/MS matching process, another routine has been introduced in the software. This routine quantifies only the MS/MS identified peptides according to the following scheme: the time profiles of the 2 major isotopes of each identified peptide are computed in a small time window where the MS/MS spectrum is recorded. Only the co-eluted signals of these 2 isotopes are analyzed to determine the peptide RT. The 3 scans averaged signal centered at this time is then compared with the full theoretical peptide isotopic pattern. This additional quantification is compared to the first one to generate a final result report. The convergence of these two quantification routines is used to improve the quantification confidence and identification coverage.

***Flow Cytometry Analysis***

U373 MG cells were incubated with increasing concentrations of Ac4ManNAz (10 to 100 µM) or vehicle for two days and then probed with 50 µM biotin phosphine for 1 h, according to the standard protocol described above. After washing, cells were subsequently stained with 5 µg/mL Streptavidin Alexa Fluor 488 conjugate in complete culture medium at 4°C for 20 min, and rinsed with PBS three times. Prior to flow cytometric analysis, cells were harvested and resuspended (1 × 10^6^ cells) in 100 µl 7-amino-actinomycin D viability dye (7-AAD) at 2.5 µg/mL in PBS. After 10 min incubation at 4°C, cells were washed with 1% BSA/PBS and then resuspended in sheath fluid before flow cytometric analysis. The cell-associated fluorescence intensity was analyzed using a BD FACSAria Flow Cytometer (Beckton Dickinson) with linear amplification of forward and side-scatter channels. Fluorescence channels 1 and 3 (FL1-A and FL3-A, respectively) were monitored using a logarithmic amplication scale. Alexa Fluor 488 and 7-AAD fluorescence signals were measured simultaneously in FL1-A and FL3-A channels, respectively. Data were analyzed using the BD FACSDiva software. Three parameters were examined for each sample: 1) the percentage of non-viable 7-AAD positive cell population which migrates up into the higher red (FL3-A) fluorescence output region of the dot plot; 2) the percentage of Alexa Fluor 488 positive cell population which is found into the higher green (FL1-A) fluorescence output region of the dot plot; 3) the Alexa Fluor 488 mean fluorescence signal associated with viable cells. Staining specificity was verified with the appropriate controls and compensation performed with single-stained samples before acquiring the multi-colored samples.

***Confocal Microscopy Analysis***

U373 MG cells (1 × 10^6^ cells) were seeded onto poly-D-lysine (10 µg/mL)-treated glass-bottom WillCo Petri dishes (Willco Wells) and grown for 6 h at 37°C and 5% CO2 in growth medium. After 6 h incubation, the medium was removed and replaced with growth medium supplemented with or without 25 µM Ac4ManNAz, and incubation was continued for another 2 days prior cells underwent Staudinger ligation with biotin phosphine (50 µM) in Hanks Balanced Salt Solution. After two washes with HBSS, cells were stained with 5 µg/mL Streptavidin Alexa Fluor 488 conjugate in HBSS at 4°C for 20 min, and rinsed twice with ice-cold HBSS. Cells were then fixed with 4% paraformaldehyde for 20 min at room temperature and subsequently washed twice with HBSS. The nucleus was counterstained for 15 min using the blue fluorescent DAPI dye (1 µg/mL in HBSS) and washed twice with HBSS. Confocal laser scan microscopy images were acquired with a C-Apochromat Zeiss 63 × /1.20 W Korr UV-VIS-IR objective using a Zeiss LSM510 Meta (Zeiss, Jena, Germany) confocal microscope with an argon/2 laser (458, 477, 488, 514 nm) and a 405 nm diode laser. Alexa Fluor 488 was imaged with the laser line 488 nm and a LP530 nm emission filter and DAPI with the 405 nm laser line and a BP 420 – 480 nm emission filter. Image acquisition and analyses were performed using Zeiss LSM 5 Pascal Confocal Microscopy Software.
